# Perceived factors influencing healthcare professionals’ adherence to the ABCDE approach: an exploratory explanatory sequential mixed-methods study

**DOI:** 10.1016/j.resplu.2026.101506

**Published:** 2026-09-24

**Authors:** Marjolein Linders, Nino H.C. Schoeber, Mathijs Binkhorst, Willem-Pieter de Boode, Cornelia R.M.G. Fluit, Marije Hogeveen

**Affiliations:** a Department of Paediatrics, Amalia Children’s Hospital, Radboud University Medical Center, Geert Grooteplein Zuid 10, 6525 GA Nijmegen, the Netherlands; b Department of Neonatology, Amalia Children’s Hospital, Radboud University Medical Center, Geert Grooteplein Zuid 10, 6525 GA Nijmegen, the Netherlands; c Radboudumc Health Academy, Radboud University Medical Center, Gerard van Swietenlaan 2, 6525 GB Nijmegen, the Netherlands

**Keywords:** ABCDE, Adherence, Emergency medicine, Critical care, Life support care, Mixed-methods research

## Abstract

**Background:**

The Airway, Breathing, Circulation, Disability, and Exposure (ABCDE) approach is considered the preferred way to assess potentially critically ill patients. This study aimed to identify perceived factors influencing healthcare professionals’ adherence to the ABCDE approach.

**Methods:**

An exploratory, explanatory sequential mixed-methods study was performed, comprising an cross-sectional questionnaire phase followed by semi-structured focus groups. A total of 239 healthcare professionals (nurses, nurse practitioners, physician assistants, residents and medical specialists) from six disciplines (Anaesthesiology, Emergency Medicine, Paediatrics and the Neonatal, Paediatric and Adult Intensive Care Units) filled out a questionnaire. Questionnaire results were analysed and used as input for five semi-structured focus groups with 22 participants. The focus groups were transcribed and analysed using template analysis, complemented by identification of additional themes.

**Results:**

Perceived factors influencing adherence to the approach were categorised into five domains: guideline, individual health professional, patient, professional interactions, and incentives/resources. The importance of the ABCDE approach was affirmed, with 95.4% recognising it as their responsibility to perform the approach. Confidence in competency was high with a median rating of 90% (IQR 80–99) on a 0–100% scale. While feedback was considered valuable, 68.2% indicated it was provided rarely. Other identified factors included the ABCDE approach’s simplicity and structure, healthcare professionals’ experience, patient condition, the presence of a predefined team leader, and the role of regular education. ‘Context dependency’, ‘ABCDE as a language’ and ‘Personal value’ were identified as additional themes.

**Conclusions:**

This study identified perceived factors influencing healthcare professionals’ adherence to the ABCDE approach. Adequate application of the ABCDE approach may require continuous learning in education and clinical practice.

## Introduction

The Airway, Breathing, Circulation, Disability, and Exposure (ABCDE) approach provides a systematic framework for the primary assessment of potentially critically ill patients (Box 1). Although assessment tools vary, the fundamental principles are consistent across age groups and clinical contexts, and the approach is advocated in major life support guidelines worldwide.^1^, ^2^, ^3^, ^4^, ^5^, ^6^, ^7^ Despite this, adherence in clinical settings is variable, with reported rates ranging from 18% to 84%.^8^ Potential influencing factors –including instructional method, patient characteristics, team size, and cognitive aids– have been described but not comprehensively investigated.^9^, ^10^, ^11^, ^12^, ^13^

Box 1 **Example of the ABCDE approach.**

**A** **= Airway.**
  Patency (look, listen, feel)
**B** **= Breathing.**
  *Effort of breathing*: respiratory rate; retractions; nasal flaring; grunting; use of accessory breathing muscles; audible in- or expiratory breathing sounds.
  *Efficiency of breathing*: thoracic movement; breathing sounds on auscultation; saturation.
  *Effect on other organs*: colour; heart rate; level of consciousness.
**C** **= Circulation**.
  *Circulation assessment:* heart rate; blood pressure; capillary refill time; pulsations.
  *Effect on other organs*: respiratory rate and depth; colour; skin temperature; level of consciousness; urinary production.
**D** **= Disability**.
  *Neurological evaluation:* AVPU-score; pupils (size, response to light); tone; glucose level.
**E** **= Exposure**.
  Body temperature; skin abnormalities.

Adapted from Linders M, Binkhorst M, Draaisma JMT, van Heijst AFJ, Hogeveen M. Adherence to the ABCDE approach in relation to the method of instruction: a randomised controlled simulation study. BMC Emergency Medicine 2021;21:121. Licensed under CC BY 4.0 (https://creativecommons.org/licenses/by/4.0/). Adapted with minor changes to sub-item selection and wording.

Adherence to any clinical guideline is shaped by a complex interplay of factors that are not uniform across contexts.^14^, ^15^, ^16^ Users may lack familiarity, have insufficient knowledge, or struggle to transfer skills acquired to clinical practice.^15^, ^17^, ^18^, ^19^ Flottorp et al. developed a validated implementation framework that groups determinants of practice into seven domains: *guideline factors, individual health professional factors, patient factors, professional interactions, incentives and resources, capacity for organisational change* and *social, political and legal factors*.^15^

No study has yet comprehensively explored factors influencing adherence to the ABCDE approach. Identifying these factors could guide the development of targeted interventions to improve adherence to the ABCDE in practice. Therefore, this study aimed to identify perceived factors influencing healthcare professionals’ adherence to the ABCDE approach, using the framework of Flottorp et al.^15^

## Materials and methods

### Study design

An exploratory, explanatory sequential mixed-methods study was conducted comprising an initial cross-sectional questionnaire phase followed by semi-structured focus groups to explore the reasons underlying the quantitative findings.^20^, ^21^ Questionnaire data, including open-ended responses, were fully analysed prior to finalisation of the focus group topic guide and before the first focus group, and directly informed the selection of areas for further exploration. Focus groups were chosen over individual interviews, because adherence to the ABCDE approach inherently involves multiple healthcare professionals, and group interaction creates contextually relevant data.^22^

### Setting

This study was conducted at the Radboud University Medical Centre (Radboudumc), Nijmegen, the Netherlands. Six disciplines regularly involved in the assessment of potentially unstable patients participated: Anaesthesiology, Paediatrics, Emergency Medicine (EM), and the Neonatal (NICU), Paediatric (PICU), and Adult Intensive Care Units (ICU), together employing 954 healthcare professionals.

### Participants

All nurses, nurse practitioners (NPs), physician assistants (PAs), residents (physicians undergoing postgraduate specialty training) and medical specialists (physicians who have completed postgraduate specialty training) within these disciplines were eligible and approached by email using convenience sampling.^23^ Participants were excluded if they had no clinical exposure to critically ill patients. Participation was voluntary, questionnaire responses were anonymous and focus groups data were anonymised post hoc. Informed consent was obtained from all participants. The institutional review board considered the study exempt from formal ethical approval (file number 2018-4993).

### Research and expert team

The research team designed and conducted the study. This team was formed by ML (Medical Doctor (MD), doctor of Philosophy (PhD) candidate), NS (MD), CF (MD, PhD, professor in medical education with workplace learning as area of special interest) and MH (MD, neonatologist, PhD, professor, and project leader of this study). An expert team of eight discipline representatives with recognised expertise in the ABCDE approach contributed to questionnaire development, alongside an external expert in both paediatric emergency medicine and medical education.

### Data collection – Questionnaire

The questionnaire comprised background data and questions on ABCDE implementation, personal attitudes, and contextual facilitators, using five-point Likert scales, multiple-choice, and open-ended formats (Supplementary material 1). It was developed by the research team and iteratively reviewed by the expert team for content relevance, acceptability and clarity, but was not formally pilot tested, cognitively tested, or assessed for internal consistency. The questionnaire was administered via Castor EDC (version 2019.1.8 (Castor EDC, Amsterdam, the Netherlands) between March 19 and April 28, 2019. Automated reminders were sent after 10 and 21 days.

### Data collection – Focus groups

Two investigators (NS, MH) developed the interview guide, which was reviewed and revised by two co-investigators (MB, CF) (Supplementary material 2). The guide was developed according to Association for Medical Education in Europe (AMEE) focus group guidelines^22^ and based on the domains described by Flottorp et al. (Table 1).^15^ The foundation of the interview guide was established a priori, before analysis of the questionnaire data, and subsequently refined based on the questionnaire findings to guide further exploration in the focus groups. This refinement was completed before the first focus group was conducted. Domains 6 (capacity for organisational change) and 7 (social, political and legal factors) were excluded primarily due to time constraints within the 40–60 min focus group format, to allow sufficient depth on the remaining five domains considered most proximal to individual and team-level adherence. The interview guide was not pilot tested.

**Table 1:** Domains Flottorp et al.^15^

**Domain**
1. Guideline factors *(e.g. clarity, feasibility and compatibility)*
2. Individual health professional factors *(e.g. knowledge and skills, cognition and behaviour)*
3. Patient factors *(e.g. patient needs, beliefs and behaviour)*
4. Professional interactions *(e.g. communication, team processes and referral processes)*
5. Incentives and resources *(e.g. availability, continuing education system and assistance)*
6. Capacity for organisational change *(e.g. authority, regulations and monitoring)*
7. Social, political and legal factors *(e.g. economic constraints, contracts and legislation)*

Five focus groups comprising three to six participants were conducted at the Radboudumc in May 2019. Sessions lasted 40–60 min, were moderated by MH (experienced in focus group facilitation), and attended only by participants and researchers (MH and NS). All profession categories and disciplines were represented in the focus groups, with 2–4 profession categories and 2–5 disciplines per group (Fig. 1, Supplementary material 3). All participants were informed about the background of the interviewer (MH) and aim of the study. MH was acquainted with some of the participants through her years working in this hospital. To minimise bias, the research team's own hypotheses and interpretations were not shared with focus group participants. However, selected, summarised questionnaire results —including feedback frequency and ward-round use of the approach— were presented to participants during the sessions to probe their reflections on these findings, consistent with the explanatory sequential design.

**Fig. 1:**
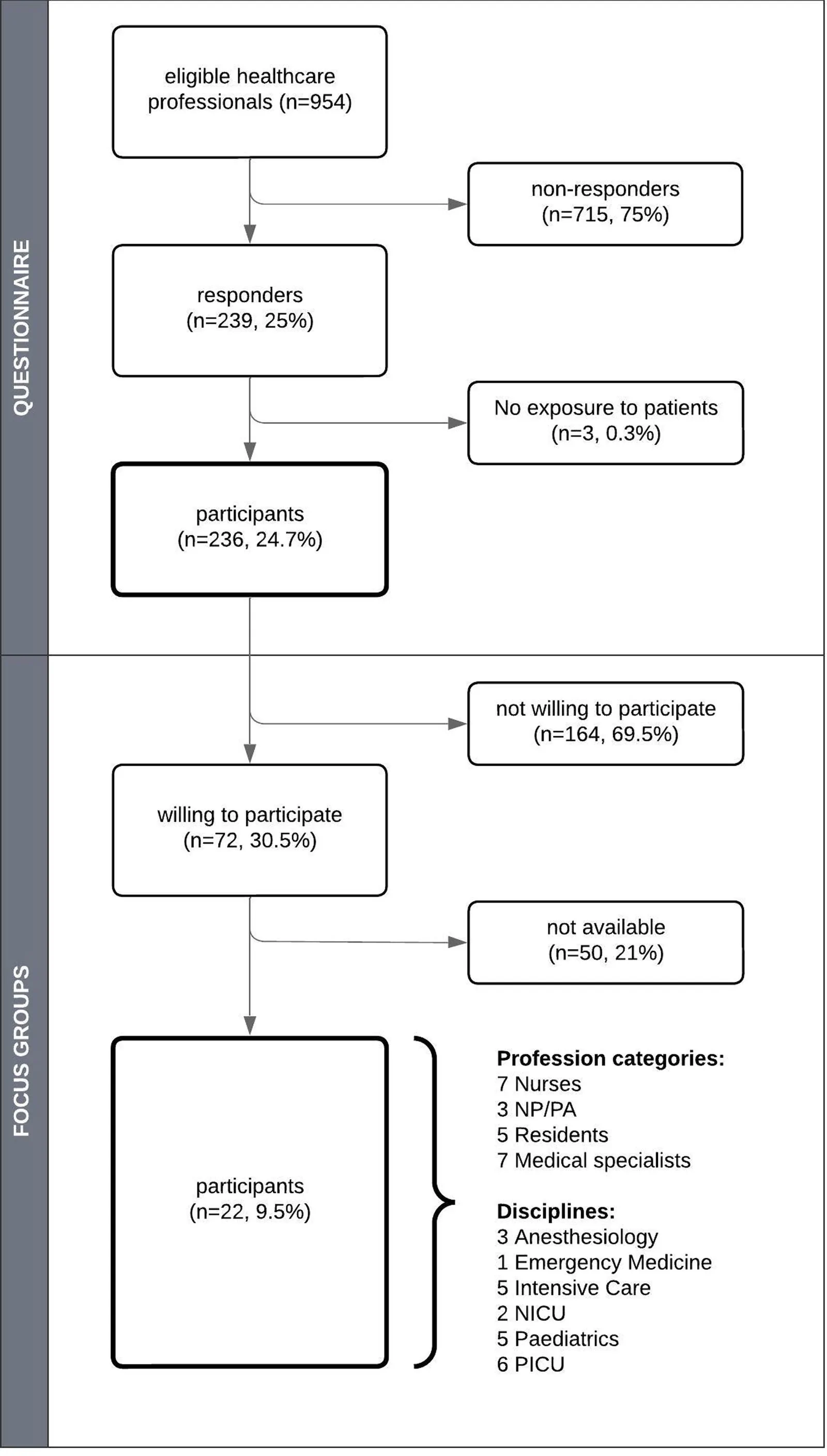
**Flow chart of participant inclusion**. NP, nurse practitioner; PA, physician assistant; NICU, neonatal intensive care unit; PICU, paediatric intensive care unit.

Sessions were audio-recorded, transcribed verbatim, and recordings subsequently deleted. Transcripts were not returned to participants, but the preliminary findings were presented to participants in a summary presentation, followed by an open discussion in which participants were invited to provide feedback on the findings (member check). This did not lead to substantive changes. Data collection continued until no substantively new codes emerged relative to the existing coding template (data saturation).^24^ This point was reached by the third focus group, with no additional codes identified in the fourth and fifth groups, confirming that the five focus groups initially planned were sufficient.

### Analysis – questionnaire

Questionnaire data were analysed descriptively before start of the focus groups using IBM SPSS version 25 (IBM Corporation, New York, USA), and open-ended responses were categorised by frequency. Kruskal Wallis tests and correlation analyses were performed as secondary analyses to explore differences and associations between questionnaire outcomes. A *p* < 0.05 was considered statistically significant. As no a priori power analysis was conducted, these inferential results should be interpreted with caution. Descriptive findings, including the categorised open-ended responses, were used to inform the focus group topic guide and to guide further exploration during the qualitative phase.

### Analysis – focus groups

Focus group transcripts were analysed using template analysis.^25^ The initial coding template, derived from five domains of the Flottorp framework,^15^ was applied by two investigators (ML, NS), who independently conducted line-by-line coding of all transcripts. Their coding was then compared, and codes that did not fit the existing template were provisionally labelled. Discrepancies were resolved through discussion with MH and CF until consensus was reached. These discussions helped interpret the data and identify recurring, substantive patterns not captured by the Flottorp domains. These were retained as inductive themes resulting in the three additional themes reported (Supplementary material 4). Analyses therefore combined deductive coding within the predefined framework with inductive identification of emergent themes. Primary outcomes were perceived barriers and facilitators influencing healthcare professionals’ adherence to the ABCDE approach. ATLAS.ti, version 8.3.20 was used (ATLAS.ti Scientific Software Development GmbH, Berlin, Germany).

### Integration of findings

Following separate analyses of the quantitative and qualitative data, NS, ML and MH jointly integrated the findings narratively using a weaving approach, bringing the quantitative findings and qualitative themes together to explore areas of convergence, complementarity, and divergence.^20^ The findings were considered theme-by-theme to assess whether the qualitative findings confirmed, explained, expanded upon, or contrasted with the questionnaire results. These comparisons were discussed with LF to reach consensus on the interpretation of the integrated findings.

## Results

Of 954 invited healthcare professionals, 239 returned completed questionnaires (25.1%); three were excluded for lack of clinical exposure, yielding 236 participants. Median age was 37 years (IQR 31–52), median clinical experience 14 years (IQR 7–27) and 178 (75%) were female. Background characteristics are provided in Table 2 and Supplementary material 5. Of the 236 participants, 22 took part in the focus groups (Fig. 1).

**Table 2:** Background characteristics.

	**Participants**			**Hospital**
	***n***	**/*N***	**%**	Distribution per discipline*(%)
*Total participants*	236		100%	

***Profession category***				
Nurse	147	/236	62%	
Nurse practitioner/Physician assistant	9	/236	4%	
Resident	36	/236	15%	
Medical specialist	44	/236	19%	

***Discipline*** ***Profession category per discipline***				
Emergency Medicine	**21**	**/236**	**9%**	
Nurses	13	/21	62%	78%
Nurse practitioner/Physician assistant	0	/21	0%	2%
Residents	7	/21	33%	9%
Medical specialists	1	/21	5%	11%

Anaesthesiology	**29**	**/236**	**12%**	
Nurses	3	/29	10.5%	28%
Nurse practitioner/Physician assistant	3	/29	10.5%	5%
Residents	9	/29	31%	33.5%
Medical specialists	14	/29	48%	33.5%

Paediatrics	**38**	**/236**	**16%**	
Nurses	26	/38	68%	49%
Nurse practitioner/Physician assistant	1	/38	3%	5%
Residents	4	/38	11%	22%
Medical specialists	7	/38	18%	24%

Intensive Care Unit	**80**	**/236**	**34%**	
Nurses	63	/80	79%	77%
Nurse practitioner/Physician assistant	1	/80	1%	2%
Residents	8	/80	10%	9%
Medical specialists	8	/80	10%	12%

Neonatal Intensive Care Unit	**35**	**/236**	**15%**	
Nurses	23	/35	66%	79%
Nurse practitioner/Physician assistant	3	/35	8.5%	5%
Residents	3	/35	8.5%	7%
Medical specialists	6	/35	17%	9%

Paediatric Intensive Care Unit	**33**	**/236**	**14%**	
Nurses	19	/33	58%	72%
Nurse practitioner/Physician assistant	1	/33	3%	2%
Residents	5	/33	15%	10%
Medical specialists	8	/33	24%	16%

Residents; physicians undergoing postgraduate specialty training.

Medical specialists; physicians who have completed postgraduate specialty training.

* Distribution of employees per profession category per discipline in the participating hospital (Radboudumc).

The importance of the ABCDE approach was affirmed by 97.9% of questionnaire respondents, a finding consistent with focus group discussions. Perceived influencing factors mapped onto five of the seven domains of Flottorp (Table 1),^15^ with three additional themes identified inductively: (1) *Context dependency*, (2) *ABCDE as a language* and (3) *Personal value*. Key findings are summarised below; illustrative quotes are presented in Table 3.

**Table 3:** Quotes related to themes and categories.

**Theme**	**Category**	**Codes**	**Illustrative quotes**
Guideline factors	Clarity	Indicates urgency, simplicity, structure, understand each other, uniformity	*“Uniformity and structure, actually. Those are two very important things, I think. And simplicity.”* (medical specialist, focus group 1) *“It’s a means rather than an end in itself. It’s a means aiming to pay attention, as you put it, to treating first what kills first. So paying attention to what is the main thing in treating someone.”* (nurse, focus group 1)
	Feasibility	Differences in interpretation, easy to learn, easy to manage, no diagnosis, non acute patients, quick conclusions, rigidity	*“You can teach anyone ABCDE. If you introduce it early on in their training and then continue to build on it, you can expand it beautifully. That would also apply to nurses. Everyone has their own layer, and the further you get, the more interventions, the more you can do with it. But the foundation remains the same.”* (resident, focus group 3)
	Compatibility	Patient’s condition	*“It’s fine that everyone actually concurs, that we all agree that we should be doing an ABCD for people in such acute situations. There’s no discussion about that at all. But in situations that are more or less chronic, where you know the patient and both of you know the patient, well, it seems a little overdone sometimes. So if you’re looking for a disadvantage… I’d never tell anyone not to do it, for it’s kind of proper for them to do it, I believe, but deep down that’s what I think.”* (medical specialist, focus group 1)
	Effort	Little effort, quick	*“What I think is another great advantage is that you can do it very quickly. You can do it very elaborately, but in itself it’s very quick.”* (resident, focus group 2)
	Other	Don’t overlook, helps to focus, difference in purpose, safety	*“I think it’s also an advantage that you treat first what’s the most urgent. So there’s already a kind of priority there.”* (medical specialist, focus group 2) *“One is structure, two is the same framework and three is safety. So if anything goes wrong, you can say, ‘let’s go back to A’. So everyone knows that safety is crucial for the team. That’s been built into this sequence.”* (medical specialist, focus group 4)
Individual health care professional factors	Cognition	Intention, self-efficacy	*“Practice makes perfect. (*…*) In five years’ time, you’ll be thinking ‘right, I can do this and I feel confident’. I thinks it’s a case of doing it over and over again, getting lots of practice. There should be plenty of scope for doing that.”* (resident, focus group 1)
	Emotions	Responsibility	*“What matters to me is whether I’m responsible or not. If a patient is handed over to me and my colleague tells me, ‘Right, I’ll be off. I have…This is the problem; I’ve consulted the supervisor; this is the diagnosis; this is the plan; he’s off to the ward now’, fine, I’m not going to check things all over again. But if they say, ‘This is the diagnosis, this is the plan; you’ve just got to check these lab results and chat with the boss’, I’m going to do it all over myself.”* (resident, focus group 3)
	Knowledge and skills	Level of experience	*“It depends*… *There’s your level of experience, I suppose. If you don’t have a lot of experience, you might be more inclined to stick to structure because it helps to approach things in a structured fashion. I know that I can speed things up by grasping a number of things in one go, by observation, whatever. So I already know or I’ve already realised that A and B are not the most important now. I need to go straight to C, and in terms of the team process, I’m skipping A and B to deal with the main issue immediately.”* (medical specialist, focus group 5)
	Professional behaviour	Emergency setting, distractions	*“What might make a difference or what might prevent you from going through the complete sequence is if there’s a major distraction, in D, for instance. That’s when you see that it’s harder for people to go through ABCDE. Or at least this is what I see happening in the emergency department. While you know you should… take a look at A and B. But that’s when I see people having the most difficulty going through the whole thing.” (*resident, focus group 2)
Patient factors		Assessment based on patient status, patient behaviour	*“I think it also makes a difference whether you’re checking on a patient on the ICU on an ordinary day or whether you’re dealing with an acute problem on the emergency department, for the structure of your ABC and the depth to which…you’re handling B, for instance. Might make a huge difference. (…) So I think it’s patient-related factors again that also contribute to how extensively you’re going through your ABCDE.”* (resident, focus group 1)
Professional interactions	Communication and influence	Previous assessment, feedback, involving others	*“When you’re in that room with several people and there’s a problem, for example, you’ll try to run through the entire ABCD as a real team: so at A there’s this, please add, who’s got any further observations, what are we overlooking. (…) So I think it’s stimulating if you’re in this together and that you offer scope to others to supplement so that this vision… (…) In the end, there’s only one leader but you’re all part of the team and everyone can complement each other. If you keep an open mind, I think that will help to obtain more relevant information.”* (resident*,* focus group 3) *“For me, getting feedback would definitely help to make me use it even more often. I sometimes feel that I’m the only one in my department who likes to use it. I know I’m not the only one; the doctors like it too, but it would help to encourage my fellow nurses.”* (nurse, focus group 1)
	Referral processes	Amount of information, clarity, knowing patient’s status	*“Well, it would certainly help to safeguard the entire system if we always did our patient handoffs in the ABCD way. The point is that the ABCD absolutely helps to structure the handoff process and the reporting because you won’t forget anything. I think that’s important.”* (medical specialist, focus group 4)
	Team processes	Atmosphere, setting an example, clear tasks, who is responsible, experience, interruptions, involve everybody, silent cockpit, team size, trust	*“If you’ve got a single structure for doing things, that helps the team performance.”* (medical specialist, focus group 5) *“The team atmosphere matters a lot too. (*…*) We just don’t have a culture in which … in which you … If you have a red PRESS [Paediatric Risk Evaluation and Stratification System], it’s compulsory for us now to do the handoff at the patient’s bedside. But it’s just not happening. Or not always, let me put it that way, and if it happens, it’s done, well, not fully in line with the ABC. It just depends on the colleague you happen to be working with whether you do or you don’t, and on the group of people that’s present in the coffee room during patient handoffs whether you’ll do it or not. Doesn’t make any sense, of course. My decision to do the handoff of my child at the bedside in line with the ABC shouldn’t be depending on the team. But it is.”* (nurse, focus group 1)
Incentives and resources	Assistance for clinicians	Tools	*“Yes, and the reports are also in line with the ABCD, so that’s a real change for the nurses. It’s made it a lot clearer, I think, what you’re expected to mention where. So that’s been a real improvement, I think, over the past few years.”* (nurse, focus group 5)
	Availability resources	Available time	*“We offer little opportunity to students to reason according to the ABC… I’ve just finished the online program myself. Well, in the entire program I was asked perhaps three times by a colleague to run through the ABC. It was actually always me who proposed doing it. And that’s just… And then, if I said I wanted to do it, they’d say ‘yeah yeah, we’ll try and do it later on, shall we’, and then it just didn’t get done.”* (nurse, focus group 1)
	Continuing Education system	Content training, education of students, frequency of training, importance of education, simulation training	*“I feel that, if you do it more often, you’ll learn to master it. In anaesthetics we often have simulations, which are super useful. We’re just doing dry runs, of course, but they raise my heartbeat anyway. They are so exciting. When you hear this beep…so that’s very instructive indeed.”* (resident, focus group 1) *“We don’t get a lot of that [simulation training]. I think… Cause you all get one of these scenario training things. Well, we don’t, never.”* (nurse, focus group 1)
Context dependency			*“It’s a bit of a problem sometimes when you’re in another setting and you find yourself in a situation and you hadn’t anticipated how you were going to do it. So you haven’t prepared and you’re thinking, hang on a minute, I’m now in a setting in which I should actually be doing an ABCDE assessment. Then it might be inconvenient first to put the team together and then to discuss this ABCD, you know, ‘Listen everyone, we’re now in this setting, and here’s what we must do’. So the early stages can be awkward sometimes.”* (resident, focus group 3)
ABCDE as a Language			*“You’re speaking the same language, so it’s easier to understand each other and respond. And supplement. Because it’s easier to see what someone…to understand what someone is doing. And to pick up on it.”* (medical specialist, focus group 5)
Personal value			*“I think that’s my main task as a nurse. And if I wasn’t allowed to do that, that would take all the fun out of it.”* (nurse, focus group 1)

### Domain: guideline factors

The simplicity, structure and cross-professional uniformity of the ABCDE approach were consistently valued, facilitating learning, mutual understanding, and a sense of safety in acute situations. Participants described multiple purposes for the approach –severity estimation, prioritisation of findings, hypothesis confirmation– and emphasised that application without a clear purpose was considered meaningless. Perceived limitations included rigidity of structure, risk of premature conclusions, and variable interpretation of findings between professionals. The approach was widely regarded as less applicable in non-acute settings.

### Domain: individual health professional factors

Most respondents considered application of the ABCDE approach their personal responsibility (95.4%). Median self-rated confidence in applying the ABCDE was 90% (IQR 80–99%), with consistently high scores across all profession categories (all ≥87%) and disciplines (all ≥80%) (Supplementary material 5). Emergency Medicine reported the highest confidence levels, significantly higher than the NICU (*p* = 0.013) and Paediatrics (*p* = 0.032). Confidence was also weakly but significantly correlated with clinical experience (Spearman's rho = −0.134, *p* = 0.046) and time since last ABCDE training (Spearman's rho = −0.145, *p* = 0.041), with higher confidence associated with fewer years of experience and more recent training (Supplementary material 5). Focus group participants suggested that performing the ABCDE may increase self-efficacy, and that less experienced professionals tend to adhere more strictly to the approach, whilst more experienced clinicians may deviate from it earlier –potentially reflecting pattern recognition rather than non-adherence. Distractions, including interruptions and sudden changes in patient condition, were identified as barriers to complete and sequential performance of the ABCDE.

### Domain: patient factors

Questionnaire respondents reported applying the ABCDE approach regularly to always across clinical contexts, including patient assessment (98.2%), electronic medical record documentation (94.5%), patient handovers (92.7%), ward rounds (88.6%), interdepartmental consultations (87.2%) and telephone consultations (86.4%) (Supplementary material 5). Focus groups findings indicated that actual use was strongly determined by patient acuity and clinical context. Use increased when a patient was evidently critically ill or injured, when the condition of a patient in critical condition changed, or in response to family concern, and decreased during ward rounds (in contrast to the questionnaire findings), in stable patient transfers, or when a recent assessment had been performed. A notable difficulty was initiating the ABCDE approach when a patient deteriorated unexpectedly from an initially non-critical condition.

### Domain: professional interactions

Professional role was considered the primary determinant of responsibility for performing the approach compared to ABCDE proficiency or patient familiarity. Residents were most often cited as responsible (89.8%), followed by nurses (75.8%) and medical specialists (66.5%) (Supplementary material 5). Focus group participants expressed varied views on task allocation, with some advocating situation-specific consensus.

A predefined team leader, clear task distribution, and adherence to a “sterile cockpit” principle (team members do not speak except for urgent matters) were consistently identified as facilitators of structured ABCDE assessment. Conversely, an unsafe team atmosphere, inappropriate team size, multiple interruptions, chaos, and lack of structure were cited as barriers. Role modelling was repeatedly emphasised as important.

The approach was also valued as a communication tool during handovers and patient transfers, though participants cautioned against information overload when every domain of the ABCDE is reported in full. The likelihood of repeating a colleague’s assessment was inversely related to the perceived experience of the initial assessor. Another reason to repeat the ABCDE approach was to obtain a personal impression to facilitate the follow-up of a patient.

Despite the recognised value of feedback, 68.2% of questionnaire respondents reported receiving it rarely or never in daily clinical practice, consistent with the focus group results (Supplementary material 5).

### Domain: incentives and resources

Most respondents had received formal ABCDE education (89.9%), with a median interval of eight months since last training. Departmental facilitation of the ABCDE approach was reported by 88%, though focus group participants frequently stated that training opportunities were insufficient in frequency, particularly for staff with infrequent exposure to critically ill patients. Simulation-based training was identified as the most valued educational modality. Repetitive, contextualised practice –including real-time application in clinical settings– was considered essential for skill acquisition and retention. Tools such as a pocket card and electronic smart phrases were infrequently used (26.3%) but widely endorsed in focus group discussions.

### Additional theme: context dependency

Application of the ABCDE approach was strongly context-dependent, shaped by professional role and discipline, level of experience, team composition and culture, departmental atmosphere, and the specific clinical situation. These contextual factors interact dynamically, making adherence variable even amongst motivated professionals.

### Additional theme: ABCDE as a language

Participants described the ABCDE approach as a shared professional language enabling mutual understanding and a tool for structured communication across disciplines, not as a goal in itself. However, variation in definitions, preferred level of explicitness, and contextual scope –for instance, whether the ABCDE constitutes a complete assessment or the first step in a broader clinical process– generates miscommunication and confusion in some contexts.

### Additional theme: personal value

Beyond its clinical utility, participants attributed personal meaning to competent performance of the ABCDE approach, linking it to professional autonomy, self-efficacy, job satisfaction, and a sense of team belonging.

## Discussion

This mixed-methods study identified a broad range of perceived factors influencing healthcare professionals’ adherence to the ABCDE approach, mapped across five of the seven domains of Flottorp et al., with three additional inductively derived themes: ‘Context dependency’, ‘ABCDE as a language’ and ‘Personal value’. The ABCDE approach was near-universally valued by healthcare professionals, viewed as their professional responsibility, and associated with high self-rated confidence. Key influencing factors included the ABCDE’s clarity and structure, professional experience, patient acuity, team dynamics, feedback provision, and access to regular education.

A notable discrepancy emerged between questionnaire and focus group data regarding ward round use: whilst 88.6% of questionnaire respondents reported applying the approach during ward rounds, focus group participants expressed reservations. This may reflect a conceptual distinction between using the ABCDE as a *communication framework* for discussing clinical findings versus using it as a *patient assessment tool* –a distinction with direct implications for how the approach is taught and evaluated in practice.

The difficulty reported in initiating the ABCDE approach when a patient deteriorates unexpectedly from an initially stable condition is clinically important, given that this represents precisely the scenario in which the approach should provide structure and confidence. This paradox may reflect acute stress responses that impair performance even when knowledge and skills are present,^26^, ^27^ or insufficient self-efficacy in unfamiliar contexts.^28^, ^29^ Serial ABCDE assessments may mitigate this by establishing a baseline and enabling earlier detection of deterioration. Both feedback and education are plausible mechanisms for improvement. However, these broad categories likely comprise several distinct strategies that may act through different mechanisms and warrant separate consideration: feedback provided immediately after simulation-based training differs from feedback embedded in routine clinical practice, where structured debriefing has been shown to improve outcomes after real resuscitations^30^ and supports reflective consolidation of experience^31^; low-dose, high-frequency (spaced) simulation may address skill decay through repeated, distributed exposure^32^; Rapid Cycle Deliberate Practice targets specific sub-skills through focused, incremental correction^33^; and workplace-based coaching has been shown to provide real-time, contextualised guidance and improved adherence to guidelines in cardiopulmonary resuscitation, suggesting potential applicability to ABCDE training.^34^ These strategies primarily target the acquisition and consolidation of skills through repeated, feedback-rich exposure, and plausibly act via the self-efficacy pathway.^28^, ^29^ Whether they also mitigate acute stress reactivity implicated in the initiation paradox^26^, ^27^ is less clear, and stress-reducing interventions during emergency care remain an important, comparatively underexplored avenue.^35^ Which of these strategies, alone or in combination, would be most effectively in improving ABCDE initiation in the specific context of acute, unexpected patient deterioration remains an important question for future research.

Concerning professional interactions, the tendency of a second assessor to repeat the ABCDE approach when the first is less experienced likely reflects competence trust, which is shaped not only by experience but also by their relationship, attitudes, and context of both professionals.^36^ Besides verification and early detection of changes in the patient’s condition, such repetition offers opportunities for observational learning –a clinically underutilised educational mechanism.

The three additional themes –context dependency, ABCDE as a language, and personal value– extend beyond Flottorp’s implementation framework and collectively suggest that adherence is not simply a matter of knowledge or motivation, but involves active meaning-making within a social and professional context. The variation in how professionals conceptualise and apply the approach —including differences in scope, explicitness and purpose— represents a form of disciplinary 'dialect' that may impede communication across teams. This finding resonates with the concept of shared mental models in team science: effective team performance depends not only on individual competence but on team members holding a sufficiently overlapping understanding of the task, roles, and shared language. Simulation-based training that explicitly builds shared mental models has been shown to enhance the quality of resuscitation.^37^ Our findings suggest that ABCDE 'dialects' may represent a specific, previously undescribed threat to this shared understanding in acute care teams. Adapting the ABCDE approach across different professional and clinical contexts, and navigating these dialects, reflects processes consistent with 'boundary crossing' learning theory^38^ and adaptive expertise.^39^ These insights have direct relevance to interprofessional education and practice: making disciplinary dialects explicit —for example through structured team debriefing or interprofessional simulation training that surfaces and reconciles differing interpretations of the ABCDE approach— may strengthen shared mental models and communication across professional boundaries, and represents a concrete target for future team-based training interventions.

This study is the first to map perceived factors influencing ABCDE adherence across disciplines and professional categories. Prior work has addressed discrete factors such as cognitive aids, teaching methods, and team leadership, in isolation.^9^, ^10^, ^11^, ^12^, ^40^ Comparison with guideline adherence studies in adjacent settings (pre-hospital, undergraduate) reveal overlapping themes, including the importance of cognitive aids, experiential learning, and structural clarity, guidelines, and safety, whilst factors such as limited protocol awareness were not applicable here, given that the ABCDE approach was universally acknowledged by our participants.^41^, ^42^, ^43^, ^44^

### Strengths and limitations

The multidisciplinary design, inclusion of multiple professional categories, and sequential mixed-methods approach are key strengths, enabling integration of quantitative and qualitative data and improving the depth and reliability of findings. The questionnaire development process, AMEE-guided interview structure, and multi-investigator approach further support validity.

The primary limitation is the questionnaire response rate of 25%, below the reported healthcare professional average of approximately 50%, though rates below 30% are not uncommon in this literature.^45^, ^46^, ^47^ Email-based recruitment without personalisation or incentives likely contributed to the response rate.^48^ Participation bias cannot be excluded; professionals with a particular affinity for the ABCDE approach may be over-represented in both the questionnaire and focus group samples. Additionally, although the questionnaire was reviewed by the expert team for content relevance and clarity, it was not formally pilot tested, cognitively tested, or assessed for internal consistency prior to distribution, which may have affected item clarity, interpretability, or measurement precision for some respondents. The absence of an a priori power calculation means inferential statistics should be interpreted cautiously (Supplementary material 5).

Regarding the focus groups, the moderator’s (MH) familiarity with some participants may have facilitated rapport and openness, but may also have influenced participants’ responses, for example through social desirability or reluctance to discuss potentially critical views. This potential influence was considered during analysis, which was based on anonymised transcripts and included an analyst (ML) who was not present during the focus groups. Data saturation was achieved after three focus groups, suggesting that the main themes had been captured, although the possibility of additional themes cannot be excluded.^24^ Finally, the decision to exclude two Flottorp domains (capacity for organisational change; social, political and legal factors) may have narrowed the analytic lens. In particular, our findings cannot speak to organisational capacity for change as defined by Flottorp et al., such as leadership authority to implement change or formal policy and monitoring mechanisms, which remains an unexplored dimension of implementation. Future research could explore these domains to assess whether extending the framework would provide additional insights.

A further limitation is the time elapsed since data collection in 2019. Although many of the identified barriers and facilitators are likely to remain relevant, changes in acute care, simulation-based education, digital documentation, workforce pressures, and interprofessional working since then may have affected some factors influencing ABCDE adherence. Finally, single-centre recruitment limits generalisability, though the fundamental principles of the ABCDE approach are consistent across comparable healthcare systems, and the identified factors are likely to be broadly relevant.

## Conclusions

This study provides a comprehensive overview of healthcare professionals’ perceived barriers and facilitators influencing adherence to the ABCDE approach, structured using the implementation framework of Flottorp et al.,^15^ and complemented by three additional themes. Adequate and consistent application may require a continuous learning process with sustained attention to both education and clinical practice. Our findings provide insights into perceived barriers and facilitators related to the algorithm itself, the professional, the patient, the team and incentives and resources. These findings suggest potential areas for intervention, including structured feedback, low-dose high-frequency simulation training, and team-based approaches that address non-technical skills (*e.g.* communication, leadership, and decision-making). Future research should examine whether these perceived factors are associated with observed ABCDE adherence in clinical practice, how interventions targeting these factors affect the transfer of skills from training environments to clinical practice, and how contextual and interprofessional factors shape adherence across different healthcare systems.

## CRediT authorship contribution statement

**Marjolein Linders:** Writing – review & editing, Writing – original draft, Project administration, Methodology, Investigation, Formal analysis, Data curation, Conceptualization. **Nino H.C. Schoeber:** Writing – review & editing, Writing – original draft, Project administration, Methodology, Investigation, Formal analysis, Data curation, Conceptualization. **Mathijs Binkhorst:** Writing – review & editing, Formal analysis, Conceptualization. **Willem-Pieter de Boode:** Writing – review & editing, Conceptualization. **Cornelia R.M.G. Fluit:** Writing – review & editing, Supervision, Methodology, Formal analysis, Conceptualization. **Marije Hogeveen:** Writing – review & editing, Supervision, Project administration, Methodology, Investigation, Formal analysis, Data curation, Conceptualization.

## Ethics approval and consent to participate

This study was performed in accordance The Code of Ethics of the World Medical Association (Declaration of Helsinki). The Institutional Review Board of the Radboudumc considered the study exempt from formal approval (file number: 2018-4993), since participants were not exposed to medical interventions. Informed consent was obtained from all participants.

## Availability of data and materials

Data gathered and analysed in this study that has not been included in this article, is available from the corresponding author on reasonable request.

## Declaration of competing interest

The authors declare the following financial interests/personal relationships which may be considered as potential competing interests: Marije Hogeveen is national course director of the Newborn Advanced Life Support course by the Dutch Foundation for the Emergency Medical Care of Children, member of the medical advisory board of the Dutch Resuscitation Council, member of ILCOR NLS taskforce, and Science Chair of the Science and Education Committee of Newborn Life Support of the European Resuscitation Council. Willem-Pieter de Boode is president of the European Society for Paediatric Research (ESPR). The remaining authors have no conflicts of interest to disclose. This study was performed without funding.
