# Supplementary material for: Perceived factors influencing healthcare professionals’ adherence to the ABCDE approach: an exploratory explanatory sequential mixed-methods study
Source: Resusc Plus. 2026 Sep 24;32:101506. doi: 10.1016/j.resplu.2026.101506 (PMC13673031; doi:10.1016/j.resplu.2026.101506)
Supplement: Supplementary file 1 [file mmc1.pdf]

# Supplementary material 1   Content questionnaire

**Part 1: Background data**

1. What is your age? (years)

2. What is your gender?
   a. Male
   b. Female

3. How many years of clinical experiences do you have? (*if medical specialist: including residency)*

4. At which department are you currently employed?
   a. NICU (neonatal intensive care unit)
   b. PICU (paediatric intensive care unit)
   c. ICU (intensive care unit)
   d. EM (emergency medicine)
   e. Anaesthesiology
   f. Paediatrics

5. What is your profession category?
   a. Nurse
   b. Nurse practitioner
   c. Physician Assistant
   d. Resident
   e. Fellow
   f. Medical specialist

6. Have you previously been working in other hospitals or institutions where you treated critically ill patients? If yes, where?

7. Have you ever received education about the ABCDE approach?
   a. Yes → 7.1 en 7.2
   b. No

7.1 How many months ago was your last ABCDE course / training?

7.2 Which course / training did you attend? (multiple answers possible)

Advanced Paediatric Life Support / Paediatric Advanced Life Support / European Paediatric Advanced Life Support / Pre-Hospital Paediatric Life Support / Advanced Cardiac Life Support / Advanced Trauma Life Support / Training by department / Theoretical lesson (presentation) / Course/training in initial education / Course/training in further education / Other

**Part 2. Implementation ABCDE approach**

1. How often is the ABCDE approach applied at your department when assessing a patient?
   1) Never
   2) Almost never
   3) Regularly
   4) Almost always
   5) Always

2. How often is the ABCDE approach applied during consultation of a patient at another department?
   1) Never
   2) Almost never
   3) Regularly
   4) Almost always
   5) Always

3. How often is the ABCDE approach used for notes in the electronic medical record at your department?
   1) Never
   2) Almost never
   3) Regularly
   4) Almost always
   5) Always

4. How often is the ABCDE approach used during (oral) handover of patient information to a colleague?
   1) Never
   2) Almost never
   3) Regularly
   4) Almost always
   5) Always

5. How often is the ABCDE approach used during the ward round?
   1) Never
   2) Almost never
   3) Regularly
   4) Almost always
   5) Always

6. How often is the ABCDE approach used in telephone consultations about a patient?
   1) Never
   2) Almost never
   3) Regularly

4) Almost always
5) Always

7. How important do you personally think that application of the ABCDE approach is in the assessment of a critically ill patient?
1) Not important at all
2) Not important
3) Neutral
4) Important
5) Very important

8. Do you consider it to be your task to perform the ABCDE approach?
1) Not at all
2) No
3) Neutral
4) Yes
5) Very much

9. Who do you think is primary responsible for the application of a complete ABCDE during the evaluation of a patient? (multiple answers possible)

Nurse / Resident (including Physician Assistant and/or Nurse Practitioner) / Medical Specialist / The one who knows the patient best / The one who masters the ABCDE structure best / The one who does not have other tasks at that moment

10. Do you receive feedback on your performance of the ABCDE approach in daily clinical practice?
1) Never
2) Almost never
3) Regularly
4) Almost always
5) Always

11. Do you use tools for the application of the ABCDE approach? E.g. pocket cards, smart phrases in the electronic medical record system et cetera.
1) Yes → 11.1
2) No

11.1. What tools?

12. Do you use algorithms, approaches and/or assessment tools other dan the ABCDE? E.g. 'short clinical impression'.
1) Yes → 12.1
2) No

12.1. Which one?

13. Does your department provide education on the ABCDE-structure?
   1) Yes → 13.1
   2) No

13.1. When, how often and is this theoretical and/or practical education? For example, only upon the start of employment or on structural basis every 3 months a practical lesson or a presentation.

14. Is using of the ABCDE approach obligatory at your department?
   1) Yes, always
   2) Yes, in specific cases → 14.1
   3) No, never

14.1. In which cases is the application obligatory?

15. Are you facilitated and/or stimulated at your workplace to apply the ABCDE approach? Think of facilitation by handing out pocket cards, posters at workplaces, stimulation by colleagues, protocols or work agreements.
   1) Yes → 15.1
   2) No

15.1. How are you facilitated or stimulated?

16. To what extent do you trust yourself in applying the ABCDE approach adequately for estimating the vital signs of a critically ill patient?

   *Scale of 'Not at all' (0.00%) to 'completely' (100.00%)*

17. Do you have any suggestions on how application of the ABCDE approach could be improved?

# Supplementary material 2 Interview guide for focus groups

**Introduction**

- Introducing interviewer and assistant
- Introduction of study
- Explanation of concept focus group interview
    - Invitation to share all ideas, opinions and experiences
    - Mainly discussion amongst participants, no right or wrong answers. The interviewer will sometimes ask in-depth questions and will guide the participants through the interview.
- Use of audio recording, anonymous transcript will be made. Recording will be erased. Signing informed consent.

**First questions**

- Introduction: Name, department, function, experience
- Open round: Think about the last time you were present during the assessment of a (potentially) critically ill patient. How was the ABCDE applied? Who performed the ABCDE approach?

**Key questions**

1. Why is the ABCDE approach important?
   Possible follow-up questions:
   a. What pros does the ABCDE approach have?
   b. What cons does the ABCDE approach have?
   c. Why do you (not) support the ABCDE approach?

2. What do you need for a complete primary survey using the ABCDE approach?
   Possible follow-up questions:
   a. What will help?
   b. What will inhibit?
   c. Are there key persons of influence? Who and why?
   d. What skills are needed?
   e. What knowledge is needed?
   f. What support is needed?
   g. What training or education is needed?
   h. Would feedback help? Why?
   i. Do emotions play a role? Which?
   j. Would adjuncts be helpful? Which?

3. What influences your choice to use the ABCDE approach?
   Possible follow-up questions:
   a. Emotions? Which?
   b. Team?

c. Previous or next assessments of the ABCDE approach?
d. Key persons of influence? Why?
e. Hierarchy?
f. Patient factors?

4. What is the influence of the team on the choice whether or not to use the ABCDE approach?
   Possible examples:
   a. Experience of other team members
   b. Trust of team members in your capabilities
   c. Use of ABCDE approach by team members
   d. Hierarchy

5. Who is best suited to apply the ABCDE approach? Why?

6. What is the relationship between your professional identity / role and the ABCDE approach?

7. What factors in your surroundings influence the adherence to the ABCDE approach?
   Examples:
   a. Is there a role for EPIC (electronic patient record)?
   b. What is the role of continuing training for the ABCDE approach? Would this be helpful? Why?
   c. What is the role of learning / working environment regarding the ABCDE approach on your adherence to it?
   d. How does the scenario setting compare to the real, clinical setting?
   e. Would other algorithms be a better choice? Which? Why?

**Ending**

- Summary
- Something that has not been asked or discussed?

## Supplementary material 3 Participants per focus group

1. 6 participants (4 profession categories, 3 disciplines)
   - Nurse | Intensive care
   - Nurse | Intensive care
   - Nurse | Paediatrics
   - Physician Assistant | Intensive Care
   - Resident | Anaesthesiology
   - Medical specialist | Intensive care

2. 4 participants (3 profession categories, 3 disciplines)
   - Nurse | Paediatrics
   - Resident | Emergency Medicine
   - Medical specialist | Paediatric Intensive Care Unit
   - Medical specialist | Paediatric Intensive Care Unit

3. 5 participants (2 profession categories, 5 disciplines)
   - Nurse practitioner | Anaesthesiology
   - Nurse practitioner | Paediatric Intensive Care Unit
   - Resident | Anaesthesiology
   - Resident | Intensive Care Unit
   - Resident | Paediatrics

4. 4 participants (2 profession categories, 2 disciplines)
   - Nurse | Neonatal Intensive Care Unit
   - Nurse | Paediatric Intensive Care Unit
   - Medical specialist | Paediatric Intensive Care Unit
   - Medical specialist | Paediatric Intensive Care Unit

5. 3 participants (2 profession categories, 2 disciplines)
   - Nurse | Paediatrics
   - Medical specialist | Paediatrics
   - Medical specialist | Neonatal Intensive Care Unit

Supplementary material 4 Code tree

| THEMATIC ANALYSIS BASED ON FLOTTORP ET AL | | |
|---|---|---|
| **GUIDELINE FACTORS** | Clarity | Indicates urgency |
| | | Simplicity |
| | | Structure |
| | | Understand each other |
| | | Uniformity |
| | Feasibility | Difference in interpretation |
| | | Easy to learn |
| | | Easy to manage |
| | | No diagnosis |
| | | Non acute patients |
| | | Quick conclusions |
| | | Rigidity |
| | Compatibility | Patient's condition |
| | Effort | Little effort |
| | | Quick |
| | Other | Don't overlook |
| | | Helps to focus |
| | | Difference in purpose |
| | | Safety |
| **INDIVIDUAL HEALTH PROFESSIONAL FACTORS** | Cognition | Intention |
| | | Self-efficacy |
| | Emotions | Responsibility |
| | Knowledge and skills | Level of experience |
| | Professional Behaviour | Emergency setting |
| | | Distractions |
| **PATIENT FACTORS** | | Assessment based on patient status |
| | | Patient behaviour |
| **PROFESSIONAL INTERACTIONS** | Communication and influence | Previous assessment |
| | | Feedback |
| | | Involving others |
| | Referral processes | Amount of information |
| | | Clarity |
| | | Knowing patient's status |
| | Team processes | Atmosphere |
| | | Setting an example |
| | | Clear Tasks |
| | | Who is responsible |
| | | Experience |
| | | Interruptions |
| | | Involve everybody |
| | | Silent cockpit |
| | | Team size |
| | | Trust |

| | | |
|---|---|---|
| **INCENTIVES AND RESOURCES** | Assistance for clinicians | Tools |
| | Availability Resources | Available Time |
| | Continuing Education system | Content training |
| | | Education of students |
| | | Frequency of training |
| | | Importance of education |
| | | Simulation training |
| **ADDITIONAL THEMES** | | |
| **CONTEXT DEPENDENCY** | | |
| **ABCDE AS A LANGUAGE** | | |
| **PERSONAL VALUE** | | |

# Supplementary material 5 Statistics questionnaire

## Part 1: Background data

1. What is your age? (years)

| | | Age (years) |
|---|---|---|
| **Total (n = 236)** | | |
| **Median** | | 37 |
| **Percentiles** | **25%** | 31 |
| | **75%** | 52 |

2. What is your gender?

| | Frequency | Percent |
|---|---|---|
| **Male** | 58 | 24.6 |
| **Female** | 178 | 75.4 |
| **Total** | 236 | 100 |

3. How many years of clinical experiences do you have? (*if medical specialist: including residency)*

| | | |
|---|---|---|
| **N** | **Valid** | 235 |
| | **Missing** | 1 |
| **Median** | | 14 |
| **Percentiles** | **25%** | 7 |
| | **75%** | 27 |

| **Profession category** | | | **Discipline** | | |
|---|---|---|---|---|---|
| | | **Years (n)** | | | **Years (n)** |
| *Nurses (n=146, missing=1)* | | | *Emergency Medicine (n=21)* | | |
| Median | | 17.0 | Median | | 8.0 |
| Percentiles | 25% | 10.0 | Percentiles | 25% | 3.5 |
| | 75% | 30.0 | | 75% | 13.0 |
| *NP / PA (n=9)* | | | *Department of Anaesthesiology (n=29)* | | |
| Median | | 20.0 | Median | | 13.0 |
| Percentiles | 25% | 10.5 | Percentiles | 25% | 7.0 |
| | 75% | 29.0 | | 75% | 19.0 |
| *Residents (n=36)* | | | *Department of Paediatrics (n=38)* | | |
| Median | | 5.0 | Median | | 11.5 |
| Percentiles | 25% | 2.6 | Percentiles | 25% | 6.0 |
| | 75% | 6.8 | | 75% | 30.0 |
| *Medical specialist (n=44)* | | | *Intensive Care Unit (n=79, missing=1)* | | |
| Median | | 16 | Median | | 15.0 |
| Percentiles | 25% | 8.5 | Percentiles | 25% | 7.0 |
| | 75% | 29.5 | | 75% | 27.0 |
| | | | *Neonatal Intensive Care Unit (n=35)* | | |
| | | | Median | | 23.0 |
| | | | Percentiles | 25% | 8.0 |
| | | | | 75% | 30.0 |
| | | | *Paediatric Intensive Care Unit (n=33)* | | |
| | | | Median | | 20.0 |
| | | | Percentiles | 25% | 11.5 |
| | | | | 75% | 35.5 |

4. At which department are you currently employed?
Table 2 in main manuscript

5. What is your profession category?
Table 2 in main manuscript

6. Have you previously been working in other hospitals or institutions where you treated critically ill patients? If yes, where?

| | **Frequency** | **Percent** |
|---|---|---|
| **Yes** | 194 | 82.2 |
| **No** | 42 | 17.8 |
| **Total** | 236 | 100.0 |

7. Have you ever received education about the ABCDE approach?

| | **Frequency** | **Percent** |
|---|---|---|
| **Yes** | 212 | 89.8 |
| **No** | 24 | 10.2 |
| **Total** | 236 | 100.0 |

| | | **Frequency** | **Percent** |
|---|---|---|---|
| **Nurse** | **Yes** | 127 | 86.4 |
| | **No** | 20 | 13.6 |
| | **Total** | 147 | 100.0 |
| **Nurse practitioner / physician assistant** | **Yes** | 8 | 88.9 |
| | **No** | 1 | 11.1 |
| | **Total** | 9 | 100.0 |
| **Resident** | **Yes** | 35 | 97.2 |
| | **No** | 1 | 2.8 |
| | **Total** | 36 | 100.0 |
| **Fellow / specialist** | **Yes** | 42 | 95.5 |
| | **No** | 2 | 4.5 |
| | **Total** | 44 | 100.0 |

7.1 How many months ago was your last ABCDE course / training?

| | | |
|---|---|---|
| **N** | **Valid** | 211 |
| | **Missing** | 25 |
| **Median** | | 8 |
| **Percentiles** | **25%** | 3 |
| | **75%** | 13 |

7.2 Which course / training did you attend? (multiple answers possible)

| | | **Frequency** | **Percent** |
|---|---|---|---|
| **Advanced Cardiac Life Support** | **Yes** | 40 | 16.9 |
| | **No** | 196 | 83.1 |
| **Advanced Paediatric Life Support** | **Yes** | 63 | 26.7 |
| | **No** | 173 | 73.3 |
| **Advanced Trauma Life Support** | **Yes** | 45 | 19.1 |
| | **No** | 191 | 80.9 |
| **Course/training in further education** | **Yes** | 96 | 40.7 |
| | **No** | 140 | 59.3 |
| **Course/training in initial education** | **Yes** | 63 | 26.7 |
| | **No** | 173 | 73.3 |

| | | | |
|---|---|---|---|
| **European Paediatric Advanced Life Support** | **Yes** | 14 | 5.9 |
| | **No** | 222 | 94.1 |
| **Paediatric Advanced Life Support** | **Yes** | 27 | 11.4 |
| | **No** | 209 | 88.6 |
| **Pre-Hospital Paediatric Life Support** | **Yes** | 6 | 2.5 |
| | **No** | 230 | 97.5 |
| **Theoretical lesson (presentation)** | **Yes** | 73 | 30.9 |
| | **No** | 163 | 69.1 |
| **Training by department** | **Yes** | 141 | 59.7 |
| | **No** | 95 | 40.3 |
| **Other *** | **Yes** | 47 | 19.9 |
| | **No** | 189 | 80.1 |

* No identical responses were given at least five times. Therefore, these answers are not included here.

## Part 2. Implementation ABCDE approach

Note: The tests that were performed besides descriptive statistics should be interpreted with caution due to the lack of an a priori power analysis.

1. How often is the ABCDE approach applied at your department when assessing a patient?

| | **Frequency** | **Percent** | **Cumulative Percent** |
|---|---|---|---|
| **Never** | 3 | 1.3 | 1.3 |
| **Almost never** | 1 | 0.4 | 1.7 |
| **Regularly** | 52 | 22.0 | 23.7 |
| **Almost always** | 86 | 36.4 | 60.2 |
| **Always** | 94 | 39.8 | 100.0 |
| **Total** | 236 | 100 | |

* Results from the Kruskal-Wallis test revealed a significant difference in the distribution of scores across the groups ($p=0.028$). However, post-hoc pairwise comparison using the Bonferroni correction did not reveal any statistically significant differences between the individual groups (all adjusted p-values $>0.05$)

** A significant difference was observed between disciplines ($p<0.001$). Post-hoc pairwise comparison, adjusted using the Bonferroni correction, indicated that paediatrics scored lower on the Likert Scale compared to the emergency department ($p=0.032$), ICU ($p<0.001$) and PICU ($p<0.001$). Additionally, the NICU had lower scores than both the PICU ($p<0.001$) and ICU ($p<0.001$).

2. How often is the ABCDE approach applied during consultation of a patient at another department?

| | **Frequency** | **Percent** | **Cumulative Percent** |
|---|---|---|---|
| **Never** | 6 | 2.5 | 2.5 |
| **Almost never** | 24 | 10.2 | 12.7 |
| **Regularly** | 65 | 27.5 | 40.3 |
| **Almost always** | 69 | 29.2 | 69.5 |
| **Always** | 72 | 30.5 | 100 |
| **Total** | 236 | 100 | |

* No significant difference was observed between profession categories

** A significant difference in distribution was observed between disciplines ($p<0.001$). Post-hoc pairwise comparison, adjusted using the Bonferroni correction, indicated that the NICU scored lower on the Likert Scale compared to the PICU ($p=0.001$) and ICU ($p=0.001$). Additionally, paediatrics had lower scores than PICU ($p=0.001$) and ICU ($p=0.002$).

3. How often is the ABCDE approach used for notes in the electronic medical record at your department?

| | Frequency | Percent | Cumulative Percent |
|---|---|---|---|
| **Never** | 3 | 1.3 | 1.3 |
| **Almost never** | 10 | 4.2 | 5.5 |
| **Regularly** | 48 | 20.3 | 25.8 |
| **Almost always** | 59 | 25.0 | 50.8 |
| **Always** | 116 | 49.2 | 100 |
| **Total** | 236 | 100 | |

* A significant difference was observed between profession categories <0.001. post-hoc pairwise comparison using the Bonferroni correction indicated significant differences between nurses and the other professional categories, showing that nurses use the ABCDE approach more frequently for this purpose.

** A significant difference in distribution was observed between disciplines (p<0.001). Post-hoc pairwise comparison, adjusted using the Bonferroni correction, indicated that Anaesthesiology uses the ABCDE approach less frequent for this purpose compared to the NICU (p=0.042), PICU (p<0.001) and ICU (p<0.001).

4. How often is the ABCDE approach used during (oral) handover of patient information to a colleague?

| | Frequency | Percent | Cumulative Percent |
|---|---|---|---|
| **Never** | 3 | 1.3 | 1.3 |
| **Almost never** | 14 | 5.9 | 7.2 |
| **Regularly** | 64 | 27.1 | 34.3 |
| **Almost always** | 82 | 34.7 | 69.1 |
| **Always** | 73 | 30.9 | 100.0 |
| **Total** | 236 | 100.0 | |

* No significant difference was found between profession categories

** A significant difference in distribution was observed between disciplines (p<0.001). Post-hoc pairwise comparison, adjusted using the Bonferroni correction, indicated that the NICU scored lower on the Likert Scale compared to the PICU (p<0.001), ICU (p<0.001), and Anaesthesiology (p=0.004). Paediatrics had lower score than the PICU (p<0.001), ICU (p<0.001), Emergency Department (p=0.017), and Anaesthesiology (p<0.001)

5. How often is the ABCDE approach used during the ward round?

| | Frequency | Percent | Cumulative Percent |
|---|---|---|---|
| **Never** | 12 | 5.1 | 5.1 |
| **Almost never** | 15 | 6.4 | 11.4 |
| **Regularly** | 36 | 15.3 | 26.7 |
| **Almost always** | 77 | 32.6 | 59.3 |
| **Always** | 96 | 40.7 | 100 |
| **Total** | 236 | 100 | |

* Significant differences were observed between profession (p<0.001). Post-hoc pairwise comparisons using Bonferroni correction indicated significant differences between nurses and the other professional categories, showing that nurses use the ABCDE approach more frequently for this purpose.

** A significant difference in distribution was observed between disciplines (p<0.001). Post-hoc pairwise comparison, adjusted using the Bonferroni correction, indicated that Anaesthesiology scored lower on the Likert Scale compared to the PICU (p=0.017) and ICU (p<0.001). The Emergency Department scored lower than the ICU (p=0.001).

6. How often is the ABCDE approach used in telephone consultations about a patient?

| | Frequency | Percent | Cumulative Percent |
|---|---|---|---|
| **Never** | 4 | 1.7 | 1.7 |
| **Almost never** | 28 | 11.9 | 13.6 |
| **Regularly** | 106 | 44.9 | 58.5 |
| **Almost always** | 76 | 32.2 | 90.7 |
| **Always** | 22 | 9.3 | 100 |
| **Total** | 236 | 100 | |

* No significant difference was found between profession categories

** A significant difference in distribution was observed between disciplines (p=0.001). Post-hoc pairwise comparison, adjusted using the Bonferroni correction, indicated that the PICU scored higher on the Likert Scale compared to the NICU (p=0.002) and Paediatrics (p=0.004).

7. How important do you personally think that application of the ABCDE approach is in the assessment of a critically ill patient?

| | Frequency | Percent | Cumulative Percent |
|---|---|---|---|
| **Not important at all** | 1 | 0.4 | 0.4 |
| **Not important** | 0 | 0.0 | 0.4 |
| **Neutral** | 4 | 1.7 | 2.1 |
| **Important** | 72 | 30.5 | 32.6 |
| **Very important** | 159 | 67.4 | 100 |
| **Total** | 236 | 100.0 | |

8. Do you consider it to be your task to perform the ABCDE approach?

| | Frequency | Percent | Cumulative Percent |
|---|---|---|---|
| **Not at all** | 1 | 0.4 | 0.4 |
| **No** | 1 | 0.4 | 0.8 |
| **Neutral** | 9 | 3.8 | 4.7 |
| **Yes** | 87 | 36.9 | 41.5 |
| **Very much** | 138 | 58.5 | 100.0 |
| **Total** | 236 | 100.0 | |

9. Who do you think is primary responsible for the application of a complete ABCDE during the evaluation of a patient? (multiple answers possible)

| | | Frequency | Percent |
|---|---|---|---|
| **Nurse** | **Yes** | 179 | 75.8 |
| | **No** | 57 | 24.2 |
| **Resident (including PA and/or NP)** | **Yes** | 212 | 89.8 |
| | **No** | 24 | 10.2 |
| **Medical specialist** | **Yes** | 157 | 66.5 |
| | **No** | 79 | 33.5 |
| **The one who knows the patient best** | **Yes** | 38 | 16.1 |
| | **No** | 198 | 83.9 |
| **The one who masters the ABCDE structure best** | **Yes** | 25 | 10.6 |
| | **No** | 211 | 89.4 |
| **The one who does not have other tasks at that moment** | **Yes** | 12 | 5.1 |
| | **No** | 224 | 94.9 |
| **Someone else *** | **Yes** | 6 | 2.5 |
| | **No** | 230 | 97.5 |

* treating physician, the leader, the one that arrives at the patient first, the one that does the handover, the intensive care specialist, subspecialists (anaesthetist, surgeon, ENT doctor etc.)

10. Do you receive feedback on your performance of the ABCDE approach in daily clinical practice?

| | Frequency | Percent | Cumulative Percent |
|---|---|---|---|
| **Never** | 31 | 13.1 | 13.1 |
| **Almost never** | 130 | 55.1 | 68.2 |
| **Regularly** | 58 | 24.6 | 92.8 |
| **Almost always** | 12 | 5.1 | 97.9 |
| **Always** | 5 | 2.1 | 100.0 |
| **Total** | 236 | 100.0 | |

11. Do you use tools for the application of the ABCDE approach? E.g. pocket cards, smart phrases in the electronic medical record system et cetera.

| | Frequency | Percent |
|---|---|---|
| **Yes** | 62 | 26.3 |
| **No** | 174 | 73.7 |
| **Total** | 236 | 100.0 |

11.1. What tools?

| Tools (alphabetic order) | Frequency * |
|---|---|
| Blood pressure monitor | 7 |
| Checklists (CRM or not specified) | 7 |
| Electronic medical record system | 12 |
| Flashlight (for pupil check) | 9 |
| Monitor | 8 |
| Pocket card (ABCDE, PEWS or EMV) | 25 |
| Stethoscope | 7 |

* Responses provided fewer than five times are not displayed here
CRM, crew resource management; PEWS, paediatric early warning score; EMV, eye opening - motor response - verbal response.

12. Do you use algorithms, approaches and/or assessment tools other dan the ABCDE? E.g. ‘short clinical impression’.

| | Frequency | Percent |
|---|---|---|
| **Yes** | 38 | 16.1 |
| **No** | 198 | 83.9 |
| **Total** | 236 | 100.0 |

12.1. Which one?

| | Frequency * |
|---|---|
| EWS (early warning scores) / PEWS (paediatric “) / MEWS (modified “) | 4 |
| RSVP (Reason, Story, Vital signs, Plan) | 11 |

* Responses provided fewer than three times are not displayed here

13. Does your department provide education on the ABCDE-structure?

| | Frequency | Percent |
|---|---|---|
| **Yes** | 176 | 74.6 |
| **No** | 60 | 25.4 |
| **Total** | 236 | 100.0 |

13.1. When, how often and is this theoretical and/or practical education? For example, only upon the start of employment or on structural basis every 3 months a practical lesson or a presentation.

| | Frequency* |
|---|---|
| **When** | |
| Part of residency program | 8 |
| Part of training program (unspecified which program) | 16 |
| In daily practice / bedside | 11 |
| | |
| **How often (answers that were given at least 5 times)** | |
| Daily | 5 |
| Every week | 5 |
| Regularly (unspecified) | 9 |
| Several times a year (unspecified) | 5 |

| | |
|---|---|
| 2 times a year | 26 |
| 1-2 times a year | 5 |
| Annually | 50 |
| Once every 2 years | 9 |
| Once every 3 years | 5 |
| | |
| **Theoretical and/or practical** | |
| Both theoretical and practical | 39 |
| Practical | 19 |
| Theoretical | 7 |
| | |
| ABCDE training | 5 |
| ALS / PALS / ATLS | 38 |
| BLS / PBLS | 12 |
| Simulation training (unspecified) | 67 |
| | |
| No answer / I don't know | 6 |

* Responses provided fewer than five times are not displayed here
ALS, advanced life support; ATLS, advanced trauma life support; BLS, basic life support; PALS, paediatric advanced life support; PBLS, paediatric basic life support.

14. Is using of the ABCDE approach obligatory at your department?

| | **Frequency** | **Percent** |
|---|---|---|
| Yes, always | 168 | 71.2 |
| Yes, in specific cases | 43 | 18.2 |
| No, never | 22 | 9.3 |
| Missing | 3 | 1.3 |
| Total | 233 | 98.7 |

14.1. In which cases is the application obligatory?

| | **Frequency*** |
|---|---|
| Acute situations / resuscitation | 11 |
| Admission of a new patient | 6 |
| Handover / transfer | 13 |
| (Potentially) critically ill patient | 15 |

* Responses provided fewer than five times are not displayed here

15. Are you facilitated and/or stimulated at your workplace to apply the ABCDE approach? Think of facilitation by handing out pocket cards, posters at workplaces, stimulation by colleagues, protocols or work agreements.

| | **Frequency** | **Percent** |
|---|---|---|
| **Yes** | 205 | 86.9 |
| **No** | 28 | 11.9 |
| **Missing** | 3 | 1.3 |
| **Total** | 236 | 100.0 |

15.1. How are you facilitated or stimulated?

| | **Frequency** |
|---|---|
| Structure of reports in electronic medical record system | 58 |
| Courses / training | 47 |
| Norm / standard / culture / uniformity | 45 |
| Handover of patient using ABCDE | 39 |
| Daily use during ward rounds | 25 |
| Address each other / providing feedback | 24 |

| | |
|---|---|
| Pocket cards | 19 |
| Assessment of patient using ABCDE | 11 |
| By colleagues / team | 10 |
| Protocols / checklists / posters | 8 |
| Repetition / continuous practicing | 6 |

16. To what extent do you trust yourself in applying the ABCDE approach adequately for estimating the vital signs of a critically ill patient?

*Scale of 'Not at all' (0.00%) to 'completely' (100.00%)*

| **N** | **Valid** | 223 |
|---|---|---|
| | **Missing** | 14 |
| **Median** | | 90 |
| **Percentiles** | **25%** | 80 |
| | **75%** | 99 |

| **Profession category** | | | **Discipline** | | |
|---|---|---|---|---|---|
| | | **%** | | | **%** |
| *Nurses (n=141, missing=6)* | | | *Emergency Medicine (n=20, missing=1)* | | |
| Median | | 90 | Median | | 94 |
| Percentiles | 25% | 80 | Percentiles | 25% | 87 |
| | 75% | 98 | | 75% | 100 |
| *NP / PA (n=9)* | | | *Department of Anaesthesiology (n=29)* | | |
| Median | | 86 | Median | | 90 |
| Percentiles | 25% | 67 | Percentiles | 25% | 80 |
| | 75% | 100 | | 75% | 97 |
| *Residents (n=34, missing=2)* | | | *Department of Paediatrics (n=35, missing=3)* | | |
| Median | | 90 | Median | | 81 |
| Percentiles | 25% | 85 | Percentiles | 25% | 75 |
| | 75% | 95 | | 75% | 95 |
| *Medical specialist (n=39, missing=5)* | | | *Intensive Care Unit (n=77, missing=3)* | | |
| Median | | 87 | Median | | 90 |
| Percentiles | 25% | 80 | Percentiles | 25% | 82 |
| | 75% | 100 | | 75% | 100 |
| | | | *NICU (n=33, missing=2)* | | |
| | | | Median | | 80 |
| | | | Percentiles | 25% | 73 |
| | | | | 75% | 95 |
| | | | *PICU (n=29, missing=4)* | | |
| | | | Median | | 90 |
| | | | Percentiles | 25% | 84 |
| | | | | 75% | 99 |

NICU, neonatal intensive care unit; PICU, paediatric intensive care unit.

## Confidence levels by profession category

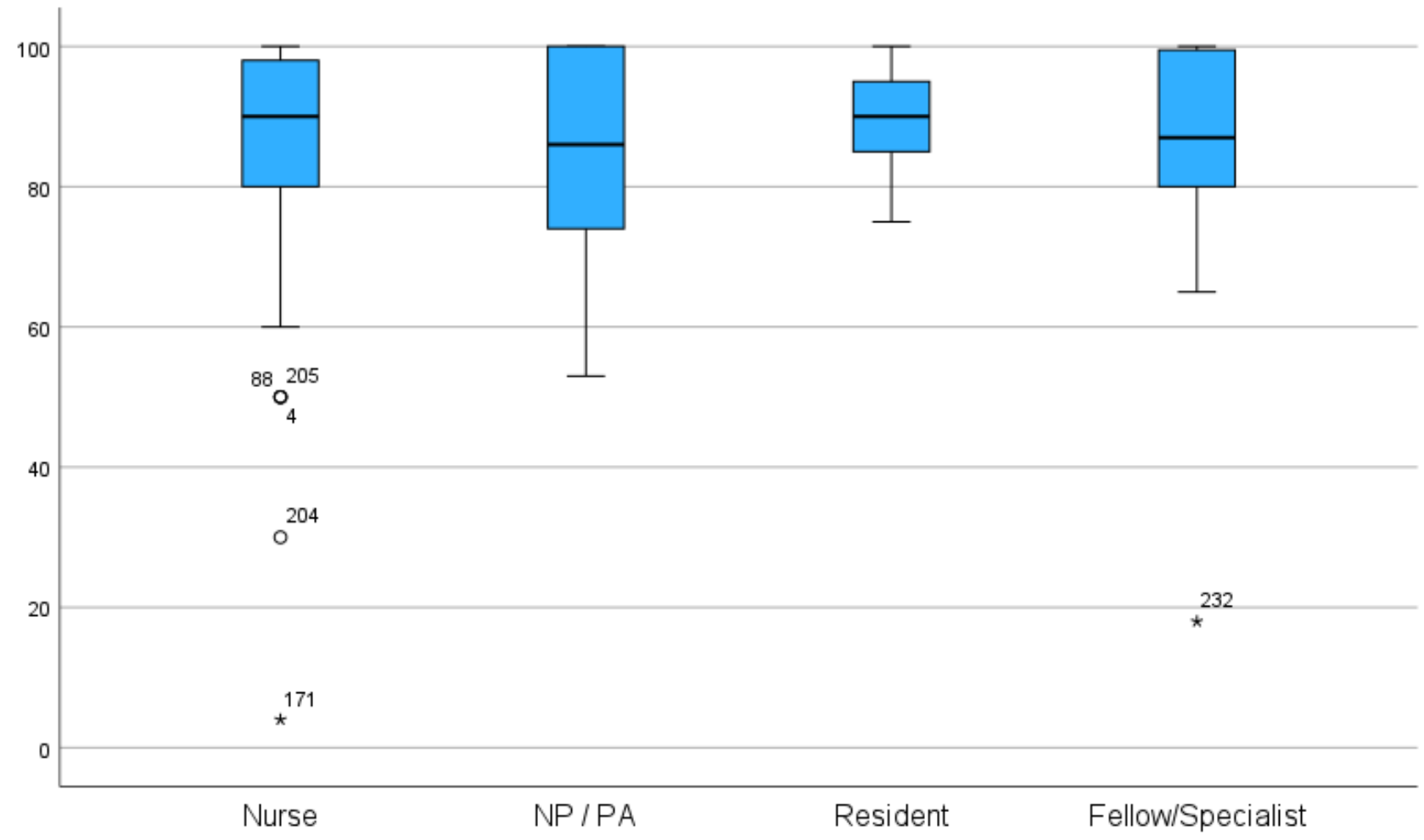


No significant differences were observed between professional categories, as determined by the Kruskal-Wallis test (p=0.723)

## Confidence levels by discipline

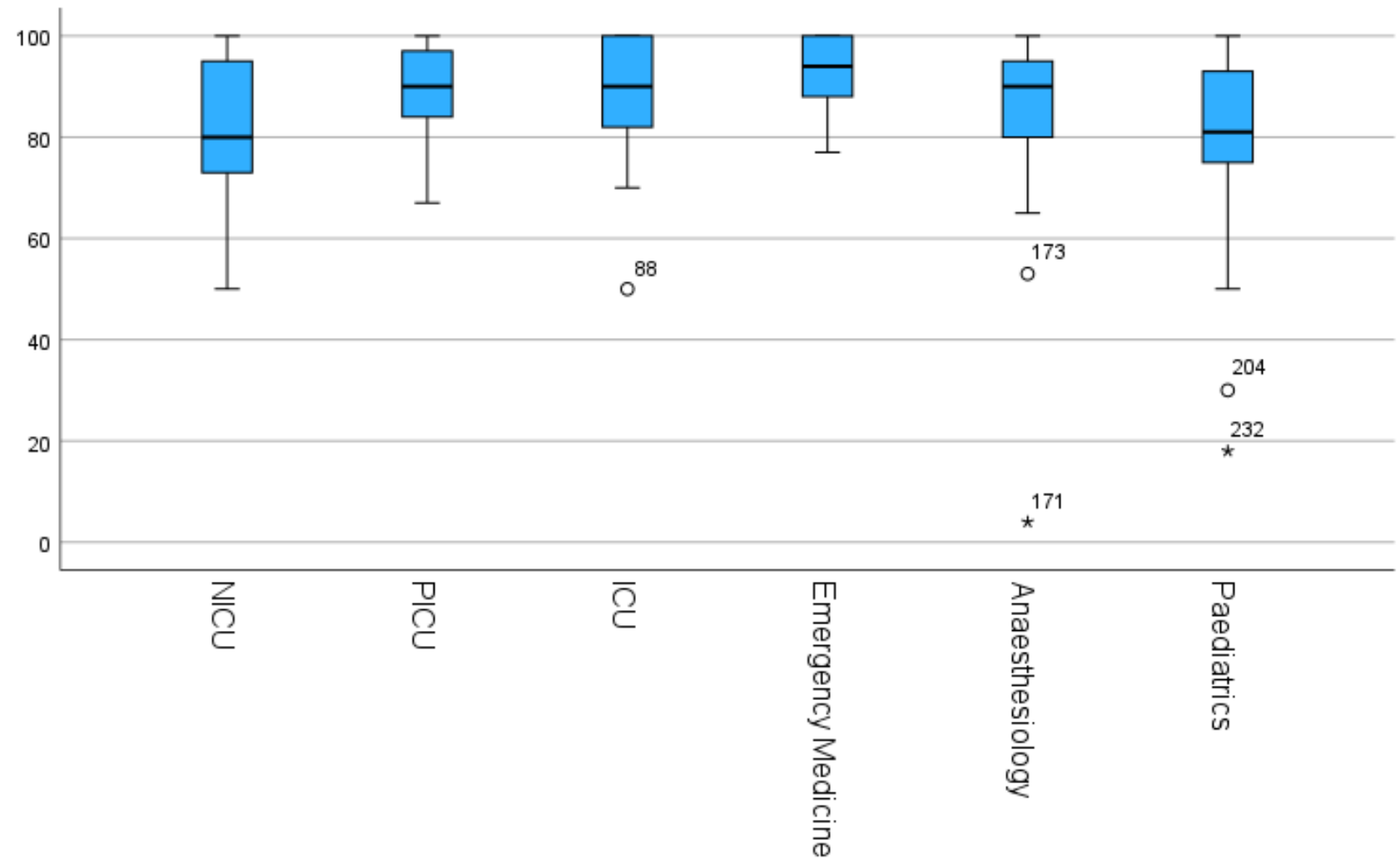


A significant difference was observed between disciplines, as determined by the Kruskal-Wallis test (=0.002), given the non-normal distribution of the data. Post hoc analysis revealed a significant difference between Emergency Medicine and NICU (p=0.013) and between Emergency Medicine and Paediatrics (p= 0.032)

## Correlation between experience and confidence level

A Spearman's rank correlation test indicated a significant association (rho = -0.134, p=0.046) between experience and confidence level, suggesting that confidence levels tend to be higher with fewer years of clinical experience.

## Correlation between education and confidence level

A Spearman's rank correlation test revealed a significant association (rho = -0.145, p=0.041) between the time since last education and confidence level, suggesting that more recent training correlates with higher confidence levels.

17. Do you have any suggestions on how application of the ABCDE approach could be improved?

| | **Frequency *** |
|---|---|
| Consistent use / daily practice | 28 |
| Training / Education | 27 |
| Addressing each other / giving feedback | 12 |
| Pocket cards / checklist / tools | 6 |

* Responses provided fewer than five times are not displayed here
